# In Situ Synthesis of MIL-100(Fe) at the Surface of Fe_3_O_4_@AC as Highly Efficient Dye Adsorbing Nanocomposite

**DOI:** 10.3390/ijms20225612

**Published:** 2019-11-09

**Authors:** Asma Hamedi, Francesco Trotta, Mahmood Borhani Zarandi, Marco Zanetti, Fabrizio Caldera, Anastasia Anceschi, Mohammad Reza Nateghi

**Affiliations:** 1 Department of Physics, Faculty of Science, Yazd University, Yazd 89195741, Iran; hamedi.asma@stu.yazd.ac.ir; 2 Department of Chemistry, University of Torino, 10125 Torino, Italy; fabrizio.caldera@unito.it (F.C.); anastasiaandrea.anceschi@unito.it (A.A.); 3 Department of Chemistry, Faculty of Science, Yazd Branch, Islamic Azad University, Yazd 8915813135, Iran; m_nateghi60@hotmail.com

**Keywords:** nanocomposite, dye adsorption, MOF, magnetic particles

## Abstract

A new magnetic nanocomposite called MIL-100(Fe) @Fe_3_O_4_@AC was synthesized by the hydrothermal method as a stable adsorbent for the removal of Rhodamine B (RhB) dye from aqueous medium. In this work, in order to increase the carbon uptake capacity, magnetic carbon was first synthesized and then the Fe_3_O_4_ was used as the iron (III) supplier to synthesize MIL-100(Fe). The size of these nanocomposite is about 30–50 nm. Compared with activated charcoal (AC) and magnetic activated charcoal (Fe_3_O_4_@AC) nanoparticles, the surface area of MIL-100(Fe) @Fe_3_O_4_@AC were eminently increased while the magnetic property of this adsorbent was decreased. The surface area of AC, Fe_3_O_4_@AC, and MIL-100(Fe) @Fe_3_O_4_@AC was 121, 351, and 620 m^2^/g, respectively. The magnetic and thermal property, chemical structure, and morphology of the MIL-100(Fe) @Fe_3_O_4_@AC were considered by vibrating sample magnetometer (VSM), thermogravimetric analysis (TGA), zeta potential, X-ray diffraction (XRD), Fourier transform infrared spectroscopy (FT-IR), scanning electron microscopy (SEM), Brunner-Emmet-Teller (BET), and transmission electron microscopy (TEM) analyses. The relatively high adsorption capacity was obtained at about 769.23 mg/g compared to other adsorbents to eliminate RhB dye from the aqueous solution within 40 min. Studies of adsorption kinetics and isotherms showed that RhB adsorption conformed the Langmuir isotherm model and the pseudo second-order kinetic model. Thermodynamic amounts depicted that the RhB adsorption was spontaneous and exothermic process. In addition, the obtained nanocomposite exhibited good reusability after several cycles. All experimental results showed that MIL-100(Fe) @Fe_3_O_4_@AC could be a prospective sorbent for the treatment of dye wastewater.

## 1. Introduction

A variety of different synthetic dyes are produced daily worldwide for use in the textile, paper, and printing industries [1,2]. The wastewater of these industries is one of the most harmful waste products due to chemicals, suspended matter, toxic compounds, and colorants [3,4]. It has been reported that in textile dyeing industries, approximately 10–25% of the color is lost during the drying process and 2–20% of the dye solution is directly discharged as wastewater [5,6]. Organic dye materials found in industrial effluents have a significant effect on the photosynthetic activity of aquatic plants by decreasing light penetration. Additionally, because of the presence of aromatics, salts and chlorides are toxic to aquatic life, causing them to die [7,8]. Rhodamin B (RhB) is a recalcitrant and cationic xanthenic dye; Due to the carcinogenic nature, the use of RhB was prohibited. However, it is often used as a colorant in the dyeing industries, food industries, and in biomedical laboratories as biological strain [9,10,11,12].

Since world supervisions have become tougher, the sewage of different industries has to be carefully examined before discharge. This has resulted in increased demand for the use of environmentally friendly technologies to remove colors from wastewater [13]. On the other hand, the introduction of an appropriate method for dye wastewater refinement is essential for environmental scientists [14]. Absorption is one of the most common methods for removing artificial dyes from aqueous sewage systems due to its simplicity, economic effectiveness, and efficiency, as well as the wide range of absorbents that can be used [15,16]. In recent years, many porous materials, including activated carbon, carbon nanotubes, graphene, and zeolites, have been made and used to remove toxic dyes from the environment [17]. However, due to the small diameter, the accessibility of the dyes to the pores of these adsorbents is limited, and the high adsorption efficiency of the dye reduces or requires longer contact time. In addition, some of these porous materials are not recyclable after use [18,19,20].

Metal–organic frameworks (MOFs) are porous substances composed of metal ions/clusters (joint) and organic ligands (linker) [21]. The metal ions/clusters attune with multi-dimensional organic ligands to compose secondary building units [22,23]. This kind of performance in metal ions and links produces MOFs with much more surface area than other porous materials such as zeolite and activated carbon [24]. These high-level areas, adjustable pore sizes, high structural flexibility and strength, easy synthesis, and high stability result in the extensive discovery of various MOFs for gas storage, molecular detection, ion exchange, absorption, sensor, imaging, catalytic, Electro catalyzing, separating, delivering medicine, photovoltaic and chemical removal [25,26,27].

Recently, MOF composites have attracted considerable attraction in various fields such as the adsorption process [22,28]. This field is partly new, and their composites were considered with different materials. To enhance the features of MOF, various types of materials such as activated carbon, Graphene oxide (GO), zero-valent iron nanoparticles (nZVI) [29], biological materials, nanofibrous membranes, etc., to increase the specific surface, higher stability, better growth of MOFs nanocrystals, and more efficient in absorbing pollutants from aquatic environments have been studied [22,30]. Activated carbon is an attractive and inexpensive option to remove organic and inorganic pollutants from water due to its high surface area, porous structure, and its thermal stability [31,32], but reactivation costs of activated carbon is high [33]. Synthesis of magnetic composites is one of the effective ways to capture these constraints due to its separation with an external magnetic field even if the solution contains a significant concentration of solids [34,35]. Therefore, it is easier to isolate contaminants and bacteria from the aquatic environment using magnetic composites [36,37].

In this work, a new magnetic MIL-100(Fe) @Fe_3_O_4_@AC nanocomposite was synthesized via two steps. Initially, Fe_3_O_4_@AC was synthesized by a chemical method at 70 °C. Next, MIL-100(Fe) was grown on the Fe_3_O_4_@AC by adding 1,3,5-Benzenetricarboxylic acid (H_3_btc) and without external source of iron at 150 °C with the hydrothermal method. Fe_3_O_4_ nanoparticles not only proceed as a shell for the AC core, but also provide iron (III) to produce MIL-100 (Fe). The MIL-100(Fe) @Fe_3_O_4_@AC was characterized using BET, TEM, XRD, SEM, FTIR, TGA, zeta potential, and VSM. The ability to remove pollutants from synthesized substances was investigated using RhB dye as pollutant. Adsorption properties including kinetics, isotherms, thermodynamics, and reusability are studied.

## 2. Results

### Characterization

**Figure 1:**
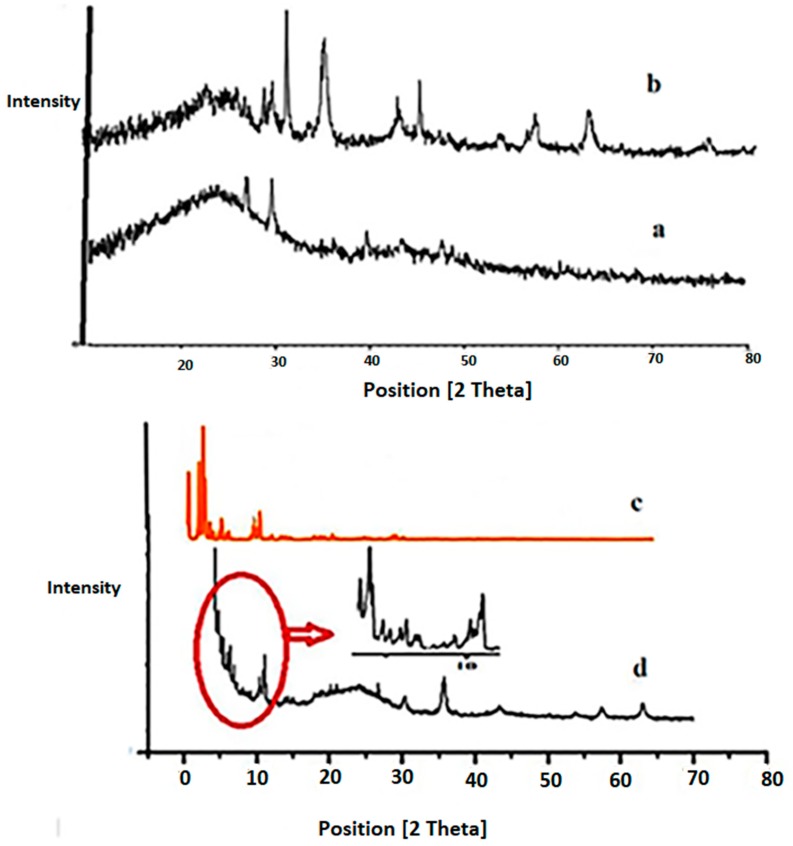
XRD pattern of (**a**) activated charcoal (AC), (**b**) Fe_3_O_4_@AC, (**c**) MIL-100(Fe)-simulated [42], and (**d**) MIL-100(Fe) @Fe_3_O_4_@AC.

The XRD patterns of AC, Fe_3_O_4_@AC nanoparticles, MIL-100(Fe)-simulated and MIL-100(Fe) @Fe_3_O_4_@AC nano-composites are shown in Figure 1. The diffraction peaks of MIL-100(Fe) @Fe_3_O_4_@AC have special peaks of each of the three absorbents AC, Fe_3_O_4_, and MIL-100 (Fe), which indicates the appropriate formation of the absorbent. The diffraction peaks at 2θ = 24°, and 26.78° are related to AC [38], while diffraction patterns at 2θ = 31.87°, 35.75°,43.41°, 57.50°, and 63° conform well with peaks of standard Fe_3_O_4_ [39,40,41]. Whereas the peaks that appeared at 2θ = 2.25°, 3.44°, 3.89°, 5.47°, 11.12°, 13.14° and 20.10° represented good agreement with already those of the simulated MIL-100(Fe) (Figure 1c) [36,42].

**Figure 2:**
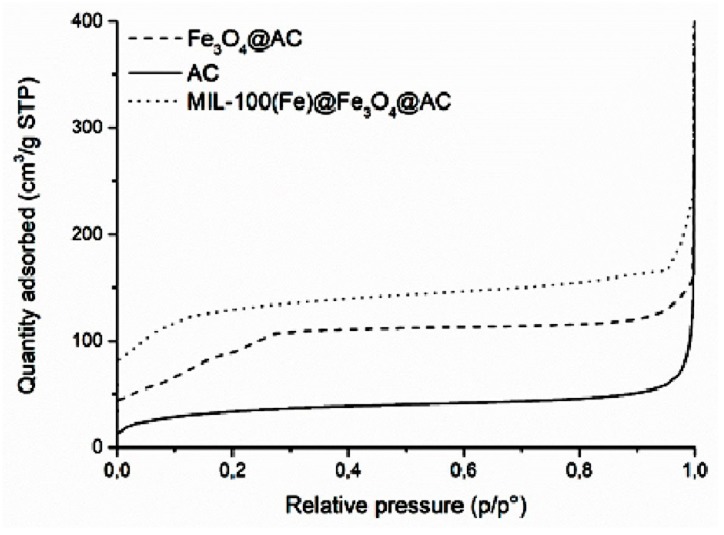
N_2_ adsorption-desorption isotherms of AC, Fe_3_O_4_@AC, and MIL-100(Fe) @Fe_3_O_4_@AC absorbents at 77 K.

The N_2_ adsorption-desorption isotherm of the AC, Fe_3_O_4_@AC and MIL-100(Fe) @Fe_3_O_4_@AC is depicted in Figure 2. The specific surface area (S_BET_) of AC, Fe_3_O_4_@AC, and MIL-100(Fe) @Fe_3_O_4_@AC were 121, 351, and 621 m^2^ g^−1^, respectively. AC, Fe_3_O_4_@AC, and MIL-100(Fe) @Fe_3_O_4_@AC showed type IV isotherms and the mesoporous structure with pore volumes of 0.0754, 0.2176, and 0.3950 cm^3^ g^−1^, respectively. The increase in the surface area of Fe_3_O_4_@AC can be attributed to the presence of Fe_3_O_4_ magnetic nanoparticles between the AC layers, increased heterogeneous surface area, and greater porosity [43,44]. Adding ligands and the formation of MIL-100(Fe) metal organic framework over the Fe_3_O_4_@AC increased the S_BET_ of MIL-100(Fe) @Fe_3_O_4_@AC until 621 m^2^g^−1^. The improved S_BET_ increased the adsorption of dye onto the absorbent surface.

**Figure 3:**
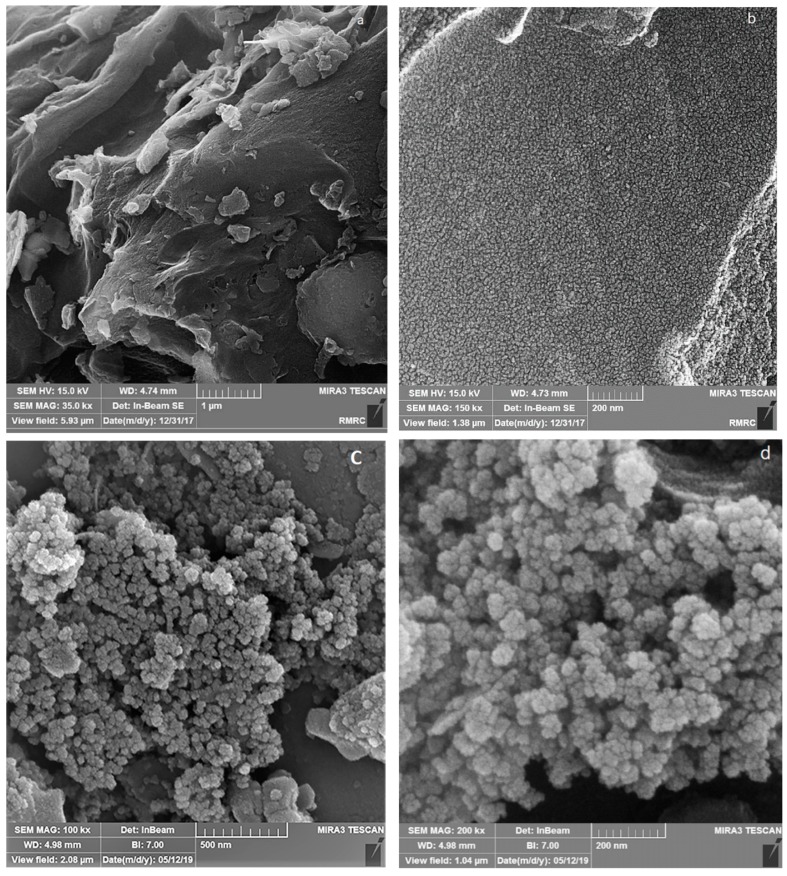

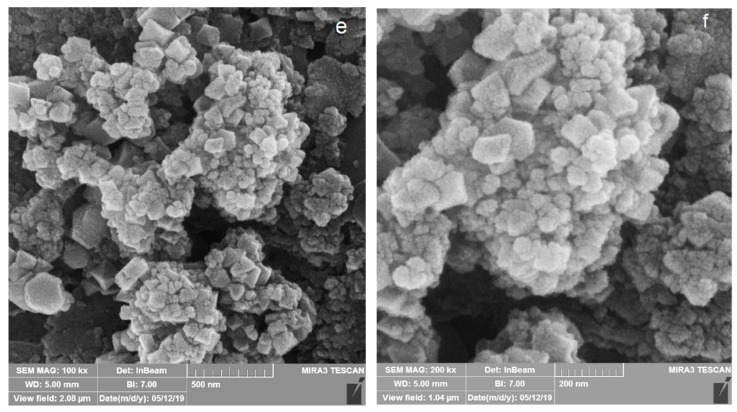
FESEM images (**a**,**b**) AC (**c**,**d**) Fe_3_O_4_@AC, and (**e**,**f**) MIL-100(Fe) @Fe_3_O_4_@AC.

**Figure 4:**
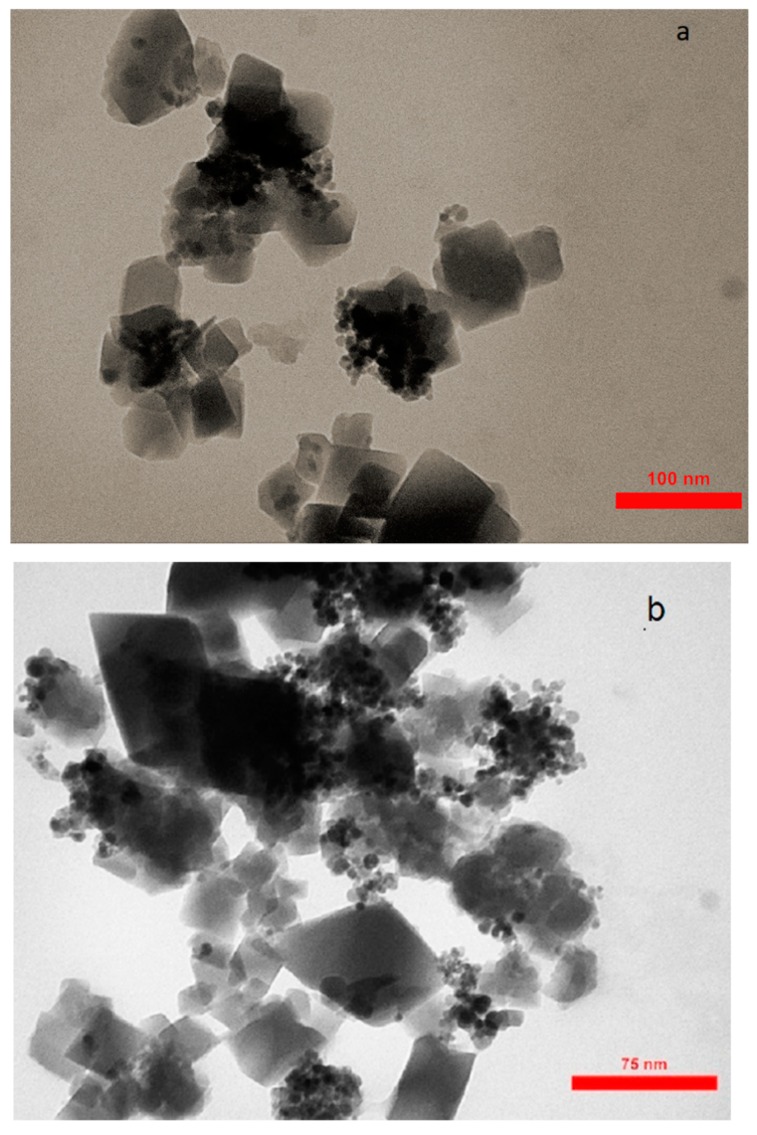

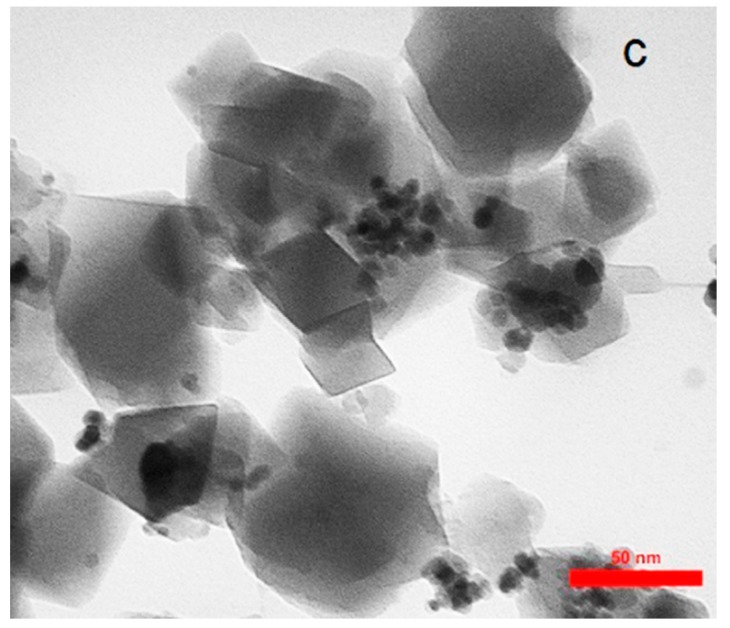
TEM images of MIL-100(Fe) @Fe_3_O_4_@AC with different magnifications (**a**) 100 nm, (**b**) 75 nm, and (**c**) 50 nm.

The FESEM of AC, Fe_3_O_4_@AC, and MIL-100(Fe) @Fe_3_O_4_@AC are shown in Figure 3 and the TEM images of MIL-100(Fe) @Fe_3_O_4_@AC with different magnifications are depicted in Figure 4. The FESEM image taken from AC (Figure 3a,b) distinguishes that nanoparticles were completely separate, granular, and the area of AC is uniform. The Fe_3_O_4_@AC spherical nanocomposite (Figure 3c,d) was found to be almost uniform in a large scale. After coating Fe_3_O_4_ on the AC surface, increased diameters, and spongy porous texture were observed [38,43], and Figure 3c,d shows that the nanoparticles of Fe_3_O_4_ agglomerated because of its magnetic interaction of Fe_3_O_4_ nanoparticles and it can be seen on the surface of AC that Fe_3_O_4_ was uniformly decorated. The particle size of Fe_3_O_4_@AC cannot be resolved by SEM, through the agglomerated and small size of the synthesized magnetic nanomaterials. In the SEM and TEM images of MIL-100(Fe) @Fe_3_O_4_@AC, the octahedral structure of MIL-100(Fe) @Fe_3_O_4_@AC nanocomposites can be seen, indicating the formation of the MIL-100(Fe) on Fe_3_O_4_@AC. The TEM images of the synthesized MIL-100(Fe) @Fe_3_O_4_@AC with different magnifications (100, 75, and 50 nm) show that the synthesized MIL-100(Fe) @Fe_3_O_4_@AC are nanomaterials with an average size of about 35-55 nm.

**Figure 5:**
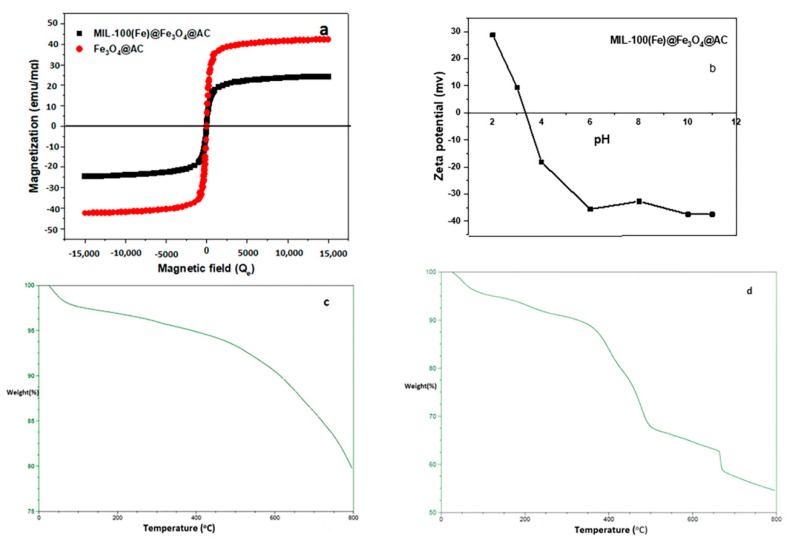
(**a**) Magnetization curves of Fe_3_O_4_@AC and MIL-100(Fe) @Fe_3_O_4_@AC, (**b**) Zeta potential trend of MIL-100(Fe) @Fe_3_O_4_@AC as a function of pH, TGA of (**c**) Fe_3_O_4_@AC, (**d**) MIL-100(Fe) @Fe_3_O_4_@AC.

Magnetic features of Fe_3_O_4_@AC and MIL-100(Fe) @Fe_3_O_4_@AC adsorbents were analyzed using a vibrating sample magnetometer in an operational magnetic field from 15 to 15 KO_e_ at room temperature. As shown in Figure 5a, the adsorbent has a paramagnetic property that it does not have any magnetic effect when removing the magnetic field. The magnetization saturation values of Fe_3_O_4_@AC and MIL-100(Fe) @Fe_3_O_4_@AC are 42.50 and 24.40 emu g^−1^, respectively. The magnetic property of the MIL-100(Fe) @Fe_3_O_4_@AC sample decreased compared to the Fe_3_O_4_@AC sample, which was due to the interaction of the H_3_btc ligand with iron present in the Fe_3_O_4_ and the formation of the MIL-100 (Fe) metal organic framework. Paramagnetic characteristics of adsorbent particles can be easily understood using a magnet. In fact, when a magnet was placed near the outer wall of a glass container containing a mixture of adsorbent particles and water (Figure 5a; inset), these particles were completely absorbed by the magnet and the dark solution was illuminated within 5 s.

The zeta potential measurement was used to evaluate the surface charge of MIL-100(Fe) @Fe_3_O_4_@AC. This is a very important indication that adsorption is used to predict the type of substrate that can be used by effective interaction with the material. Figure 5b exhibits the zeta potential trend at a pH of 2–11. According to the results, the pH_zpc_ values were achieved between 28.8 and −37.5 mV and the isoelectric point of the MIL-100(Fe) @Fe_3_O_4_@AC was realized to be nearly 3.4. It can be concluded that the adsorbent has a positive charge level in the range of 2–3.4, which makes solid interaction with materials with negative loads easier. The MIL-100(Fe) @Fe_3_O_4_@AC surface is negatively charged at pH above 3.4 (pH_solution_ > pH_pzc_) which is desirable for cationic dye adsorption [45].

Thermogravimetric analysis is one of the methods used to appoint the weight variations of a specimen as a function of temperature under a controlled atmosphere [46]. Sample thermal stability can also be remarked using TGA analysis. The TGA curves of Fe_3_O_4_@AC and MIL-100(Fe) @Fe_3_O_4_@AC in an interval of 50 to 800 °C are shown in Figure 5c,d, respectively. At an early stage, the loss of 3% and 5% total weight of Fe_3_O_4_@AC and MIL-100(Fe) @Fe_3_O_4_@AC were due to moisture up to 150 °C, respectively. Weight decrease in the first stage can be related to volatile materials (especially moisture) that adsorbed on the surface of adsorbents [44,47]. It should be noted that at this step, the weight loss of the MIL-100(Fe) @Fe_3_O_4_@AC nanocomposite sample is higher than Fe_3_O_4_@AC, which can be due to the presence of more water molecules in the nanocomposite structure. In the second step, weight loss of 2% and 5% in Fe_3_O_4_@AC and MIL-100(Fe) @Fe_3_O_4_@AC occurred in the range of 200–330 °C due to solvent removal and volatiles released by the decomposition of organic materials [7], respectively. In the third step, the main loss of 22.5% of MIL-100(Fe) @Fe_3_O_4_@AC total weight happened due to decomposition of H_3_btc molecules in the MIL-100(Fe) from 350 to 500 °C [48] and the weight of Fe_3_O_4_@AC remained constant at this temperature range. In the fourth step, weight loss of 16% and 12.5% in Fe_3_O_4_@AC and MIL-100(Fe) @Fe_3_O_4_@AC occurred in the range of 500–800 °C due to solvent removal the decomposition of organic compounds associated with Fe_3_O_4_ and polycyclic compounds decomposition [49].

**Figure 6:**
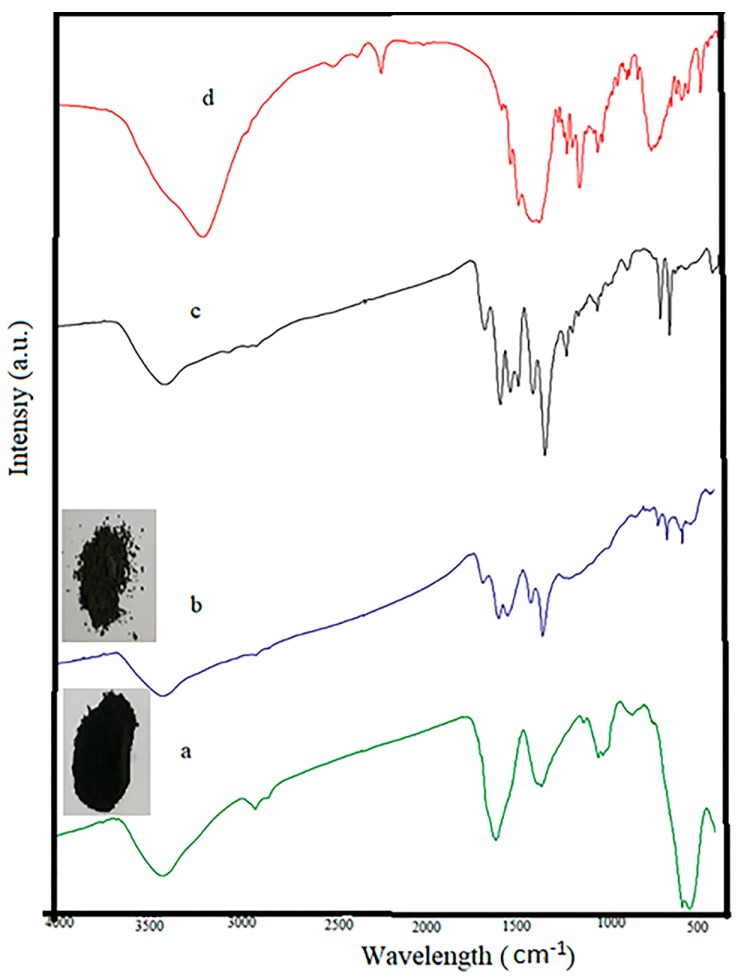
FT-IR spectra of (**a**) Fe_3_O_4_@AC, (**b**) MIL-100(Fe) @Fe_3_O_4_@AC, (**c**) Rhodamine B (RhB) loaded on MIL-100(Fe) @Fe_3_O_4_@AC and (**d**) RhB.

The FT-IR spectra of Fe_3_O_4_@AC, MIL-100(Fe) @Fe_3_O_4_@AC, RhB loaded on MIL-100(Fe) @Fe_3_O_4_@AC and RhB samples are shown in Figure 6. The FT-IR spectra of Fe_3_O_4_@AC, MIL-100(Fe) @Fe_3_O_4_@AC, RhB loaded on MIL-100(Fe) @Fe_3_O_4_@AC shows a broad absorption at 3430 cm^−1^ which is corresponding to the stretching vibrations of hydroxyl groups and H_2_O molecules [50]. Additionally, absorption at 2927 cm^−1^ is related to C–H bonds [51]. Peaks at 1382, 1631 cm^−1^ are related to O–C–O group and C=C aromatic ring vibration [52] and peaks at 1055 and 1079 cm^−1^ are attributed to presence of a C–O group and the Fe–O–C interaction, respectively [51]. Peaks at 588 and 628 cm^−1^ coincide the presence of iron oxide in the sample (Fe–O bonds) [53]. The emergence of these bands approves the formation of magnetic nanoparticles on the AC surface. The peaks of MIL-100(Fe) @Fe_3_O_4_@AC and RhB loaded on MIL-100(Fe) @Fe_3_O_4_@AC (Figure 6b,c) appeared at 1703, 1616, 760, and 711 cm^−1^ belong to stretching vibration of C=O in carboxyl groups and stretching vibration of aromatic rings in organic ligand (H_3_btc) and the peaks at 1571 and 1445 cm^−1^ were observed for MIL-100, respectively [49]. The peak intensity at 1375 cm^−1^ related to C–O stretching vibration of carboxyl groups was increased. All evidence suggests that the MIL-100(Fe) @Fe_3_O_4_@AC nanocomposite was successfully synthesized. In addition, the color difference between the black Fe_3_O_4_@AC nanoparticles and the gray MIL-100(Fe) @Fe_3_O_4_@AC nanocomposite can be considered as a good evidence for the successful synthesis of the desired composition. After adsorption of RhB, some of the peaks found in FT-IR spectrum of RhB including 648, 940, 1045, 1099, 1234, 1265, 1523, 1564, and 2381 cm^−1^ were observed at the RhB loaded on MIL-100(Fe) @Fe_3_O_4_@AC spectrum, which confirmed the RhB color absorption by MIL-100(Fe) @Fe_3_O_4_@AC absorbent.

## 3. Discussion

### 3.1. Comparison of Adsorption Capacity

**Figure 7:**
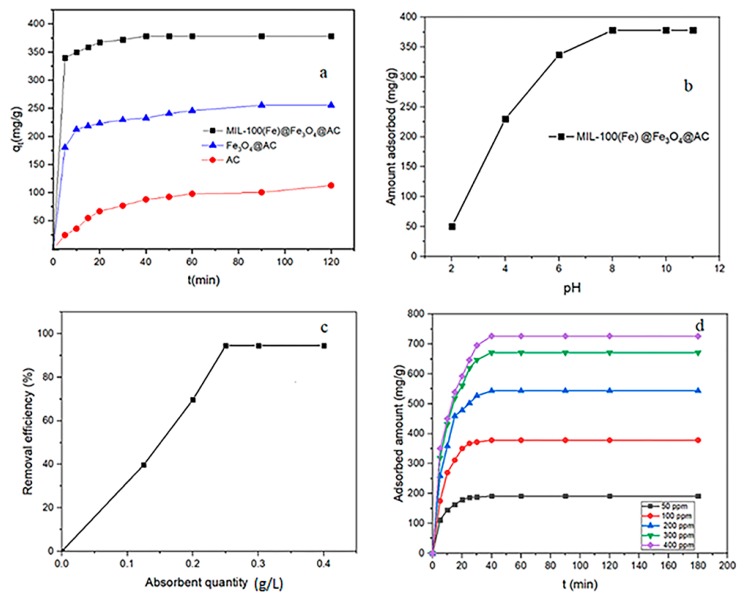
(**a**) Effect of surface modification on removal of RhB by AC, Fe_3_O_4_@AC, and MIL-100 (Fe) @Fe_3_O_4_@AC adsorbents (agitation speed = 230 rpm, adsorbent dosage = 0.25 g L^−1^, temperature = 295 K, C_0_ = 100 mg L^−1^, and pH = 8). Effect of (**b**) pH and (**c**) MIL-100(Fe) @Fe_3_O_4_@AC dose and (**d**) contact time on RhB removal.

In order to compare the absorption capacities of synthetic adsorbents, the effect of RhB adsorption on AC, Fe_3_O_4_@AC, and MIL-100(Fe) @Fe_3_O_4_@AC adsorbents was investigated. Figure 7a demonstrates that the absorption capacity of MIL-100(Fe) @Fe_3_O_4_@AC is higher than that of the other two adsorbents and also the contact time value is less. After 40 min (the optimal contact time value of MIL-100(Fe) @Fe_3_O_4_@AC, the absorption capacity of MIL-100(Fe) @Fe_3_O_4_@AC is approximately 3.4 times the absorption capacity of AC and 1.5 times that on Fe_3_O_4_@AC due to its higher specific surface area and the resulting synergistic adsorption effect.

### 3.2. Effect of pH

pH is a considerable factor in the adsorption process, stability and ionization degree of dyes, and the surface charge of adsorbent [54]. Figure 7b shows the influence of sample pH on the absorption capacity of RhB dye over MIL-100(Fe) @Fe_3_O_4_@AC in the range of 2–11 with constant initial concentration of 100 mg L^−1^ and the stirring speed of 230 rpm at 295 K. With increasing pH from 2 to 8, the RhB dye absorption capacity was increased and then remained constant up to pH 11. At very higher pH, there is electrostatic attraction between cationic RhB and OH^−^ ions. Additionally, at a very low pH (pHsolution < pHpzc), there is an electrostatic repulsion between cationic RhB and positive MIL-100(Fe) @Fe_3_O_4_@AC due to the presence of additional H^+^ ions. As in pH 2, very little absorption capacity was obtained at about 50 mg g^−1^.

### 3.3. Effect of Adsorbent Dose

The effect of different adsorbent quantities to remove RhB was surveyed from 0.125 to 0.4 g L^−1^ with the initial concentration of 100 mg L^−1^ at a constant stirring rate of 230 rpm (Figure 7c). The results show that increasing the amount of MIL-100(Fe) @Fe_3_O_4_@AC adsorbent from 0.125 to 0.25 g L^−1^ increased the removal efficiency of RhB and then remained constant afterwards. Therefore, the optimum adsorbent value was chosen 0.25 g L^−1^.

### 3.4. Effect of Contact Time

The effect of contact time at different initial concentrations of RhB (50–400 mg L^−1^) on MIL-100(Fe) @Fe_3_O_4_@AC was investigated (Figure 7d). The results showed that the adsorption of RhB on the adsorbent was rapid and absorbed about half of the dye in the first 10 min, then the adsorption rate decreased and equilibration time was achieved after 40 min. Therefore, 40 min was considered as the optimal time for further studies.

### 3.5. Adsorption Kinetics

The adsorption kinetics of RhB on MIL-100(Fe) @Fe_3_O_4_@AC at different pollutant concentrations were studied by 3 models: the pseudo first-order model, the pseudo second-order model, and intraparticle diffusion model indicated by the following formulas.

(1) $$\ln(q_{e}-q_{t})=\ln q_{e}-k_{1}t$$

(2) $$\frac{t}{q_{t}}=\frac{1}{k_{2}q_{e}^{2}}+\frac{1}{q_{e}}t$$

(3) $$q_{t}=k_{id}t^{1/2}+C$$

where *q_e_* (mg g^−1^) and *q_t_* (mg g^−1^) are the amounts of RhB adsorbed at equilibrium and random time *t* (min) respectively, and *k*_1_ (min^−1^) and *k*_2_ (g mg^−1^ min^−1^) are the constants of pseudo-first-order and the pseudo-second-order rates respectively. *k*_id_ (mg g^−1^ min^−1/2^) is the intra-particle diffusion rate constant and *C* is the intercept for the first linear phase [39,55,56].

**Figure 8:**
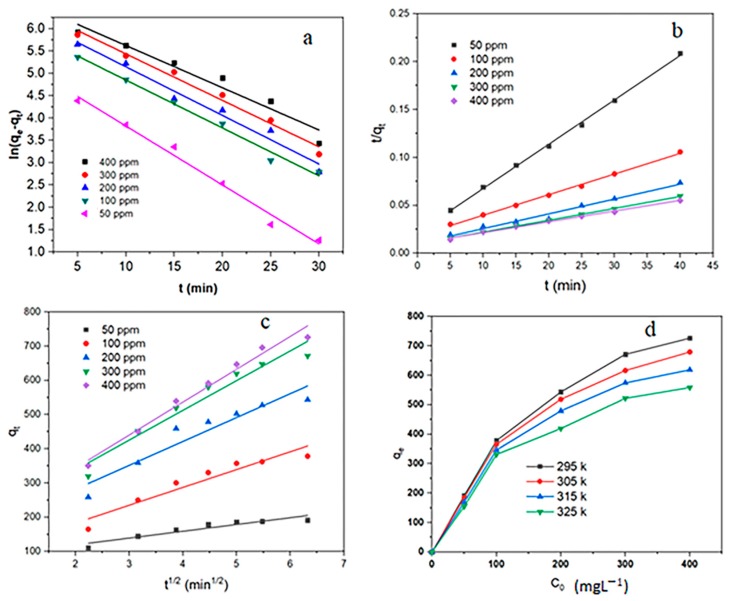
Plot of (**a**) pseudo-first-order kinetic isotherm, (**b**) pseudo-second-order kinetic isotherm, (**c**) intraparticle model, and (**d**) adsorption isotherms for the RhB adsorption on MIL-100(Fe) @Fe_3_O_4_@AC.

The kinetic parameters of the three models are given in Table 1. The results of Table 1 and Figure 8 show that the correlation coefficient quantities of the pseudo-second-order model are more than two models (R^2^ > 0.99). In addition, theoretical *q_e_* values calculated from the pseudo-second-order model were more suitable with empirical *q_e_* values. Additionally, the values of *k* in the pseudo-second-order model are much smaller than the other two models. These results indicate that the RhB adsorption on MIL-100(Fe) @Fe_3_O_4_@AC conformed with the pseudo-second-order model.

### 3.6. Adsorption Isotherms

In order to investigate the interaction mechanism of the species with the adsorbent surface during the adsorption process and also the estimation of absorption capacity, the adsorption isotherms of RhB on MIL-100(Fe) @Fe_3_O_4_@AC are shown in Figure 8d and the adsorption process of RhB with the popular Langmuir, Freundlich, and Tamkin models were compared. In the Langmuir model, the maximum adsorption occurs when the absorbent surface is completely covered by the target species and the solid surface is homogeneous. Under such conditions, monolayer adsorption takes place [57,58]. The Freundlich model considers the adsorption phenomenon as a multi-layered and heterogeneous phenomenon. It is predicted in Temkin isotherm like Freundlich isotherm that the adsorption heat decreases proportionately as the number of adsorption layers increases [59]. Langmuir, Freundlich, and Temkin models are illustrated by the following Equations (4), (5), and (6) respectively.

(4) $$\frac{C_{e}}{q_{e}}=(\frac{1}{q_{m}b})+(\frac{1}{q_{m}})C_{e}$$

(5) $$\ln q_{e}=\ln k_{f}+(\frac{1}{n})\ln C_{e}$$

(6) $$q_{e}=A\ln K_{T}+A\ln C_{e}$$

where *q_e_* (mg g^−1^) is the amount of the dye adsorbed at the equilibrium, *C_e_* is the equilibrium concentration of RhB in solution (mg L^−1^), *q_m_* is maximum adsorption capacity (mg g^−1^), *b* is the Langmuir adsorption constant (L mg^−1^), *k_f_* and *n* are the Freundlich constants related to the adsorption capacity (mg g^−1^) and adsorption intensity or surface heterogeneity, *K_T_* is the equilibrium binding constant (mol^−1^) corresponding to the maximum binding energy, and the constant *A* is related to the heat of sorption [60,61].

Figure 8d shows that the maximum RhB absorption capacity occurred at 22 °C. It announces that temperature is an important factor affecting the absorption capacity of MIL-100(Fe) @Fe_3_O_4_@AC for RhB. Additionally, the Langmuir, Freundlich, and Temkin isotherm parameters and the regression (R^2^) are summarized in Table 2. As seen, the values of Langmuir isotherm correlation coefficients were higher than the other two isotherms to absorb RhB (R^2^ > 0.99). This shows that the adsorption of RhB by the adsorbent MIL-100(Fe) @Fe_3_O_4_@AC is better described by the Langmuir model than the other two models, Freundlich and Tamkin. Therefore, there is a monolayer and homogeneous adsorption. In addition, the *q_m_* calculated by this model is very close to the actual values. Table 2 shows that the maximum adsorption capacity of MIL-100(Fe) @Fe_3_O_4_@AC is 769.23 mg/g at 295 K. In addition, the Langmuir adsorption constant for RhB decreases with increasing temperature, which indicates that RhB adsorption on MIL-100(Fe) @Fe_3_O_4_@AC is more favorable with lower temperatures.

In order to appraise the desirable adsorption system between an adsorbent and adsorbate, the separation factor is calculated from the Equation (7).

(7) $$R_{L}=\frac{1}{1+bC_{0}}$$

where *b* (L/mg) is the Langmuir constant and *C*_0_ (mg/L) is the initial dye concentration. There are four applicable adsorption methods based on the *R_L_* value: irreversible adsorption (*R_L_* = 0), favorable adsorption (0 < *R_L_* < 1), linear adsorption (*R_L_* = 1), or unfavorable adsorption (*R_L_* > 1) [62]. All quantities of *R_L_* were found to be 0–1, indicating that the adsorption of RhB on the MIL-100(Fe) @Fe_3_O_4_@AC level is desirable.

Comparison of adsorption capacity of MIL-100(Fe) @Fe_3_O_4_@AC nanocomposite with other adsorbents is presented in Table 3. It can be observed that the *q_m_* value of MIL-100(Fe) @Fe_3_O_4_@AC is about higher than that of reported adsorbents.

### 3.7. Adsorption Thermodynamics

The thermodynamics of RhB on MIL-100(Fe) @Fe_3_O_4_@AC were studied at temperature range of 295–325 K. The thermodynamic parameters such as the standard Gibbs free energy change (Δ*G°*), enthalpy change (Δ*H°*), and entropy change (Δ*S*°) were calculated using the following equations and the thermodynamic data obtained are reported in Table 4.

(8) $$K_{C}=\frac{q_{e}}{C_{e}}$$

(9) $$\Delta G^{{}^{\circ}}=-RT\ln K_{C}$$

(10) $$\ln K_{C}=\frac{\Delta S^{{}^{\circ}}}{R}-\frac{\Delta H^{{}^{\circ}}}{RT}$$

where *K_c_*, *R*, and *T* are the adsorption equilibrium constant, the universal gas constant (8.314 J mol^−1^ k^−1^), and temperature in Kelvin, respectively. The quantities of Δ*H°* and Δ*S*° were determined from the slope and intercept by plotting ln*K_c_* against 1/*T* [53,67].

According to the data in Table 3, the negative values of Δ*G*° at different temperatures show the spontaneity of the RhB absorption process. Additionally, the Δ*G*° values were lowered by a rise in temperature, which indicates that the absorption of RhB on the MIL-100(Fe) @Fe_3_O_4_@AC nanocomposite was more spontaneous at lower temperatures. Negative value of ΔH° shows that the adsorption of RhB onto MIL-100(Fe) @Fe_3_O_4_@AC is an exothermic process. In addition, the negative amount of Δ*S*° indicates a regularity increase. Based on the above-mentioned results, it can be concluded that the driving force for RhB adsorption on MIL-100(Fe) @Fe_3_O_4_@AC is managed by an enthalpy change rather than an entropy influence.

### 3.8. Recyclability of MIL-100(Fe) @Fe_3_O_4_@AC for the RhB Adsorption

**Figure 9:**
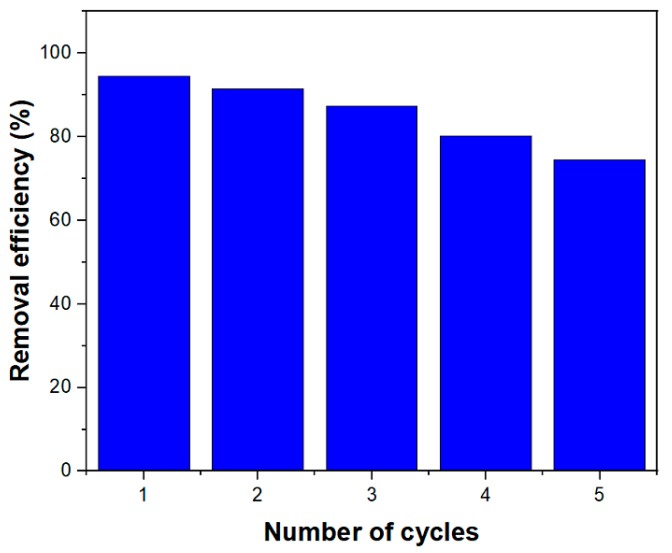
Reusability of MIL-100(Fe) @Fe_3_O_4_@AC after five cycles. (C_0_ = 100 mg/L, sorbent dosage = 0.25 g/L, and temperature = 295 K).

Recyclability is very important for industrial and functional applications. A suitable adsorbent for industrial use must have the high adsorption capacity, rapid diffusion, and excellent desorption characteristics [39]. After RhB adsorption, desorption tests were performed to evaluate MIL-100(Fe) @Fe_3_O_4_@AC reformation. Different eluents such as ethanol, acetone, 0.1 M HCl, and 0.1 M NaOH were used and acetone was the best eluent for desorption. Chemically adsorbed RhB cannot be thoroughly washed with acetone and, therefore, a marked decrease in the removal efficiency was initially seen. As shown in Figure 9, it was found that the removal efficiency of RhB was 75% after five recovery times of MIL-100(Fe) @Fe_3_O_4_@AC. Therefore, the MIL-100(Fe) @Fe_3_O_4_@AC nanocomposite is a suitable adsorbent for removing industrial organic dyes for reusability.

## 4. Materials and Methods

### 4.1. Chemicals and Materials

All reactants were obtained from commercial sources and used without further purification.

Ferric chloride hexahydrate (FeCl_3_·6H_2_O, 99 wt%, Merck, Darmstadt, Germany), Charcoal activated (AC) with a size ranging from 10 to 15 nm, Iron (II) Sulfate Heptahydrate (FeSO_4_·7H_2_O), and 1,3,5-Benzenetricarboxylic acid (H_3_btc) were purchased from Merck. RhB (C_28_H_31_ClN_2_O_3_) with molar weight 479.01 g mol^−1^ (Sigma-Aldrich, Darmstadt, Germany) were used as the adsorbates.

### 4.2. Synthesis of Fe_3_O_4_@AC Nanocomposite

**Figure 10:**
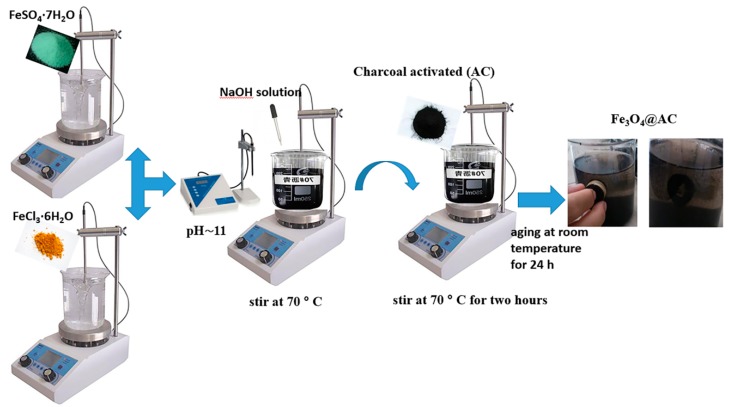
General scheme for the magnetic activated carbon (Fe_3_O_4_@AC) preparation.

Fe_3_O_4_@AC was synthesized according to the method given by Dinesh Mohan [38] with some changes (Figure 10). A total of 4 g Iron (II) Sulfate Heptahydrate in 100 mL of distilled water and 7.3 g of Iron (III) Chloride Hexahydrate in 150 mL of water were dissolved. After adding together, the mixture was rapidly stirred at 70 °C. In order to reach pH 11, a NaOH solution (1.0 M) was used. During NaOH addition, the suspension became dark brown at pH ~ 6 because of the existence Fe(OH)_3_ and Fe(OH)_2_ in the solution, and then Fe(OH)_3_ was coupled with Fe(OH)_2_ and was dehydrated to form Fe_3_O_4_ and H_2_O and the solution became black at pH ≈ 11. After adjusting the pH, 5 g of AC was added to the suspension and was stirred for 2 h. The suspension was aged at room temperature for 24 h and then repeatedly washed with deionized water and ethanol several times by an external magnet. The products were then dried at 90 °C.

### 4.3. Synthesis of MIL-100(Fe) @Fe_3_O_4_@AC

Magnetic MIL-100(Fe) @Fe_3_O_4_@AC was synthesized via a hydrothermal method reaction of 0.232 g of Fe_3_O_4_@carbon with 0.2625 g (1.25 mmol) of H_3_btc added to 30 mL of water and sonicated for 30 min. After thermal treatment in a Teflon-lined autoclave for 24 h at 150 °C, the grey reaction product was recovered by external magnet and washed 10 times with water and ethanol. Then the wet obtained solid was finally dried for 10 h at 80 °C and kept in a desiccator.

### 4.4. Characterization

FT-IR spectra in transmission mode were obtained by using a Bruker Vector 22 spectrophotometer equipped with Globar source, DTGS detector, and working with 16 scans at 4 cm^−1^ resolutions in the 4000–500 cm^−1^ range. Spectra were registered by dispersing samples in KBr pellets (1:20 wt. ratio). Thermal analyses were carried out in a TGA-TA Instruments, models Q600 (for BBS precursor) and 2950 (for BNS precursor) under either nitrogen or air atmosphere (gas flux 100 mL·min^−1^). Analyses were performed by weighing 10 mg of sample mass in an alumina open pan, following the subsequent thermal program: heating (ramp of 10 °C min^−1^) from room temperature up to 800 °C. Powder XRD patterns were obtained by means of a PW3040/60 X’Pert PRO MPD diffractometer (PANalytical, Rigaku, Japan) equipped with copper anode, working at 45 kV and 40 mA, in a Bragg–Brentano geometry. Transmission electron microscopy (TEM) was performed on a CM-120 (JEOL, Tokyo, Japan) operating at an accelerating voltage of 200 kV. Field emission scanning electron microscopy (FE-SEM) was performed using TE-SCAN (Hitachi, Tokyo, Japan). Gas-volumetric analyses were carried out performing N_2_ adsorption experiments at 77 K by means of an ASAP 2020 instrument (Micromeritics, CA, USA) to determine the specific surface area (BET model) of the samples (ca. 0.1 g) that they were previously outgassed for 12 h at 300 °C in vacuum (residual pressure 10–2 mbar) to ensure complete removal of atmospheric contaminants from the materials surface before measurements. The zeta potential of the as-synthesized core-shells were measured at different pH using Zeta Potential Analyzer (Nano-ZS ZEN3600, Malvern, UK) at room temperature. A UV–vis spectrophotometer (CE 2501, CECIL instruments, Cambridge, UK) with 1.0 cm glass cuvettes was used for the measurement of the absorbance at 558 nm for RHB. The pH of the solutions was measured with a Metrohm 780 pH meter (Metrohm Co., Herisau, Switzerland). The magnetization curve was measured at room temperature under a varying magnetic field from −15,000 to 15,000 O_e_ using a BHV-55 vibrating sample magnetometer.

### 4.5. General Approaches for the Adsorption Experiments

At first, dye solutions were provided using requisite concentrations using deionized water. The adsorbent was kept in a desiccator before use. The Batch process was performed by mixing 5 mg MIL-100(Fe) @Fe_3_O_4_@AC with 20 mL of color solution (50–400 mg/L) at the adjustable pH and constant temperature (295–325 K) in oscillating water bath shaker at 230 rpm until achieving equilibrium (typically 40 min). After adsorption, the solution was put next to the magnet for 1 min and the residual RhB in the solution was measured using UV-visible spectrophotometer at adsorption wavelengths of 558 nm. The amount of dye adsorbed on the MIL-100(Fe) @Fe_3_O_4_@AC adsorbent and Percentage removal of RhB were measured using the following equations [68]:

(11) $$q_{e}=\frac{(C_{0}-C_{e})V}{m}$$

(12) $$Efficiency(\%)=\frac{\left(C_{0}-C_{t}\right)}{C_{0}}\times 100$$

where *q*_e_ is the equilibrium adsorption capacity (mg·g^−1^); *C*_0_ and *C*_e_ are the initial and equilibrium dye concentrations (mg·L^−1^), respectively; *V* is the volume of solution (L) and *m* is the mass of the MIL-100(Fe) @Fe_3_O_4_@AC used (g) [63].

## 5. Conclusions

In this study, the magnetic nanocomposite MIL-100(Fe) @Fe_3_O_4_@AC was first synthesized with the average crystal size of −50 nm using hydrothermal method for removal RhB. Initially, in order to increase the specific surface area of AC, easier separation of AC from the aqueous medium and increase the RhB dye absorption capacity, Fe_3_O_4_@AC nanomaterials were synthesized. Then, using the H_3_btc ligand, the MIL-100(Fe) metal organic framework was formed on Fe_3_O_4_@AC with the exception that no other metal source was used and the iron present in Fe_3_O_4_ was used as the iron source. Addition of H_3_btc and formation of the MIL-100 (Fe) metal organic framework on Fe_3_O_4_@AC increased S_BET_ of MIL-100(Fe) @Fe_3_O_4_@AC to 621 m^2^/g which increased the adsorption capacity of RhB on MIL-100(Fe) @Fe_3_O_4_@AC 3.5 times larger than the adsorption capacity of RhB on AC, and 1.5 times larger than it on the Fe_3_O_4_@AC. The nanocomposites had an average size of about 35-55 nm and they were superparamagnetic, they can be separated from solution within 5 s. Adsorption analysis and kinetic experiments showed that the RhB adsorption process followed the Langmuir and pseudo-second-order isotherms, respectively. The adsorption capacity of RhB on MIL-100(Fe) @Fe_3_O_4_@AC was 769.23 mg/g, which was higher than the other adsorbents. In addition, the thermodynamic parameters indicated that the adsorption process for the RhB dye was a spontaneous and exothermic process. It had a good recyclability after five recovery times. High absorption capacity, high specific surface area, good recycling, and high magnetic properties make the MIL-100(Fe) @Fe_3_O_4_@AC nanocomposite a good candidate for textile dye removal from wastewater. We believe that this study could pave the way for the design and fabrication of new magnetic nanocomposites.

## Figures and Tables

**Table 1:** The kinetic parameters and coefficients of the pseudo-first-order, pseudo-second-order, and intraparticle models for RhB adsorption on MIL-100(Fe) @Fe_3_O_4_@AC.

Initial Conc. (mg/L)	Pseudo-First-Order Model			Pseudo-Second-Order Model			Intraparticle Diffusion Model		
	*q_e_*	*K_1_*	R^2^	*q_e_*	*K_2_*	R^2^	*k_id_*	*C*	R^2^
50	171.19	0.1321	0.9863	217.39	9.93 × 10^−4^	0.9987	19.815	79.908	0.8907
100	374.2	0.1074	0.989	454.54	2.67 × 10^−4^	0.9978	52.048	79.093	0.9155
200	511.42	0.109	0.9771	666.66	2.21 × 10^−4^	0.991	69.76	142.51	0.9036
300	651.05	0.1042	0.9854	833.33	1.48 × 10^−4^	0.9993	86.894	164.82	0.9462
400	714.73	0.0947	0.9502	909.09	1.19 × 10^−4^	0.995	95.971	152.67	0.9796

**Table 2:** Temkin, Langmuir and Freundlich constants for sorption of RB on MIL-100(Fe) @Fe_3_O_4_@AC.

T (K)	q_exp_	Langmuir			Freundlich			Temkin		
		*q_m_*	*b*	R^2^	*K_F_*	*n*	R^2^	*K_T_*	*A*	R^2^
295	726.36	769.23	0.0884	0.9923	192.09	3.9262	0.9153	3.974	105.66	0.969
305	679.42	714.28	0.0679	0.9935	153.78	3.5486	0.898	1.903	110.12	0.9663
315	618.72	666.66	0.0473	0.9951	113.39	3.1162	0.8614	0.854	116.56	0.9486
325	558.72	625	0.0336	0.9911	91.09	2.9735	0.8165	0.587	111.09	0.9099

**Table 3:** The *q_m_* values for the adsorption of RhB on different adsorbents.

Adsorbent	*q_m_* (mg g^−1^)	Reference
Fe_3_O_4_/AC	182.48	[43]
Fe_3_O_4_/MIL-100(Fe)	28.36	[10]
Fabricated magnetic lignosulfonate (MLS)	22.47	[56]
Zn-MOF	3.750	[6]
AC/CeO_2_	324.6	[9]
raw orange peel (ROP)	3.266	[63]
In-MOF@GO-2	267	[64]
Ni@MOF-74(Ni)	177.8	[65]
Cobalt sulphide nanostructures	1138	[66]
MIL-100(Fe) @Fe_3_O_4_@AC	769.23	Present study

**Table 4:** Parameters for adsorption of RhB on MIL-100 (Fe) @Fe_3_O_4_@AC. (C_0_ = 200 ppm, agitation speed = 230 rpm, adsorbent dosage = 0.25 g/L, temperature = 295–325 K).

Temp. (K)	*C_e_* (mg L ^−1^)	*q_e_* (mg g^−1^)	Δ*G*° (kJ mol^−1^)	Δ*H*° (kJ mol^−1^)	Δ*S*° (J mol^−1^ K^−1^)
295	64.1418	543.4328	−5.2408	−18.0705	−43.3417
305	70.3987	518.4052	−5.0626		
315	80.2147	479.1412	−4.5319		
325	95.2142	419.1432	−4.0046		
